# Writing and Erasing Encryption Information Based on Frustrated Lewis Pair Chemistry

**DOI:** 10.1021/prechem.3c00062

**Published:** 2023-07-28

**Authors:** Hui Fang, Xiao Long, Xia Lan, Bao-Yi Ren, Guohua Xie

**Affiliations:** † Sauvage Center for Molecular Sciences, Hubei Key Lab on Organic and Polymeric Optoelectronic Materials, Department of Chemistry, 12390 Wuhan University, Wuhan 430072, China; ‡ College of Science, 118412 Shenyang University of Chemical Technology, Shenyang 110142, China; § The Institute of Flexible Electronics (Future Technologies), Xiamen University, Xiamen 361005, China

**Keywords:** weak coordination bond, Lewis acid−base adducts, inkjet printing, luminescence, anticounterfeit

## Abstract

Lewis acid–base adducts resulting from instantaneous interactions provide a cost-effective strategy for color tuning and anticounterfeiting information. Herein, we report the construction of luminescent Lewis acid–base adducts via inkjet printing. Due to the unique weak coordination bond of B→N, it is feasible to construct anticounterfeiting information that is easy to erase. The in situ postsynthesis of the luminescent quick response codes via inkjet printing facilitates precision chemistry control to change the emission ranging from deep-blue (peaking at 407 nm) to orange-red (peaking at 597 nm). The encrypted information can be quickly erased either by modulating the temperature to dissociate the weak coordination or strong Lewis base to promote a neutralization reaction.

## Graphical abstract

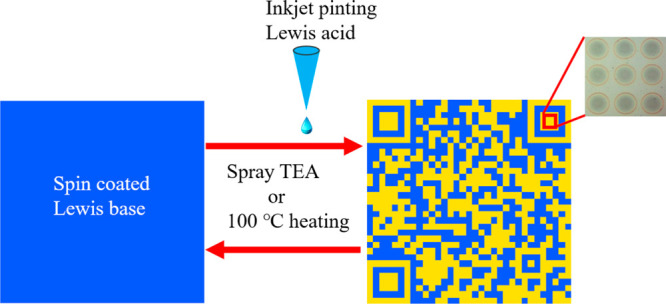

## Introduction

Counterfeit is a global problem, which poses an important threat to individuals, society, and national security.^1,2^ For example, counterfeit goods tend to be ubiquitous in daily lives, threatening life and property. The urgent need of national security and human health has urged scientists to develop reliable anticounterfeiting technology. Anticounterfeit labels based on inkjet printing technology are one of the best solutions to the above-mentioned problems. Inkjet technology can realize the fine informative patterns in a more cost-effective way.^3–5^ Additionally, the inks used for anticounterfeiting are also extremely diverse. Recently, the luminescent molecules, covering purely organic compounds, quantum dots (QDs), and perovskite materials, have been widely studied as the ink ingredients for inkjet printing.^6–9^ Organic fluorescent and phosphorescent materials have achieved great success in organic light-emitting diodes (OLED),^10–12^ Moreover, there are some candidates that are favored for information encryption and storage and anticounterfeiting.^13^

Feng’s group used two conjugated fluorescent polymers to print the quick response (QR) codes that could be used for medical identification.^14^ In the aspect of drug anticounterfeiting, Xu’s team reported the low cost up-conversion fluorescent 3D QR codes by printing them on the drug capsules, which was used to identify the authenticity of drugs.^15^ The design of anticounterfeiting labels using photochromic materials has also become popular. Bradley’s group manipulated photochromic spiropyrans and fluorophores to control Förster resonance energy transfer (FRET) between functional photochromic units (energy acceptors) and fluorophores (energy donors). The inkjet printed two-dimensional codes displayed red emission under UV irradiation (365 nm) and green color under blue light irradiation (470 nm).^16^ However, there are few studies on the erasable anticounterfeiting information. This is mainly because the luminescent patterns were environmentally and chemically stable.^8^ Therefore, the anticounterfeiting pattern cannot be changed once produced. Therefore, the encrypted information can be easily decoded. To address this issue, a frustrated Lewis pair provides an efficient and simple way to enable erasable anticounterfeiting information. Welch et al. found that adding Lewis acid to the nitrogen-containing Lewis base can significantly adjust the band gap.^17^ Furthermore, the reaction of Lewis acid–base pair is spontaneous and programmable by in situ postsynthesis of Lewis acid–base adducts.^18^ Usually, the syntheses of narrow bandgap emitters often require multiple and complicated procedures. In contrast, Lewis acid–base adducts present the simpler approaches to modulate optical properties as desired.^19^ Fluorescent molecules containing nitrogen sites, e.g., pyridyl, triazine, and pyrimidine units, might serve as electron donors to bind Lewis acids. The redistribution of the electron density between the π-conjugated system and the Lewis acid would lower the lowest unoccupied molecular orbitals (LUMOs) energy level. Meanwhile, the highest occupied molecular orbitals (HOMOs) energy level might be barely affected, which account for the reduction of the bandgap.^20^ The stronger the Lewis acidity is used, the more pronounced red-shift of absorption and emission spectra was detected.^21^ Most likely, such Lewis acid–base adducts do not form the strong covalent bonds. The weak coordination bond between Lewis acid and Lewis base seems predominant.^22^ The interaction of a Lewis acid and base have been extensively applied in the activation of H_2_ and small molecules, primarily as catalysts for various reactions.^23–26^ However, their applications in anticounterfeiting have been explored clearly.

The previous designs of inkjet printing luminescent molecules for anticounterfeiting information employed the nonemissive Lewis acid to encrypt the information.^27^ In this contribution, we developed a new approach to quickly write and erase the luminescent Lewis acid–base adducts for advanced anticounterfeiting.

Herein, we investigated the interactions of a new acceptor–donor–acceptor molecule synthesized in our laboratory with the Lewis acid tris­(pentafluorophenyl)­borane (BCF). The deep-blue emissive Lewis base *p*TRZSX featured two symmetric diphenyltriazine acceptors and a spiro­[fluorene-9,9′-xanthene] donor (shown in Figure 1). By printing the BCF ink on the seeding film with *p*TRZSX, the reaction occurred quickly in situ to form the luminescent Lewis acid–base adduct. It is worth noting that the anticounterfeiting patterns can be easily removed by another strong Lewis base. In addition, the anticounterfeiting patterns can be simply erased by heating the printed film.

**Figure 1:**
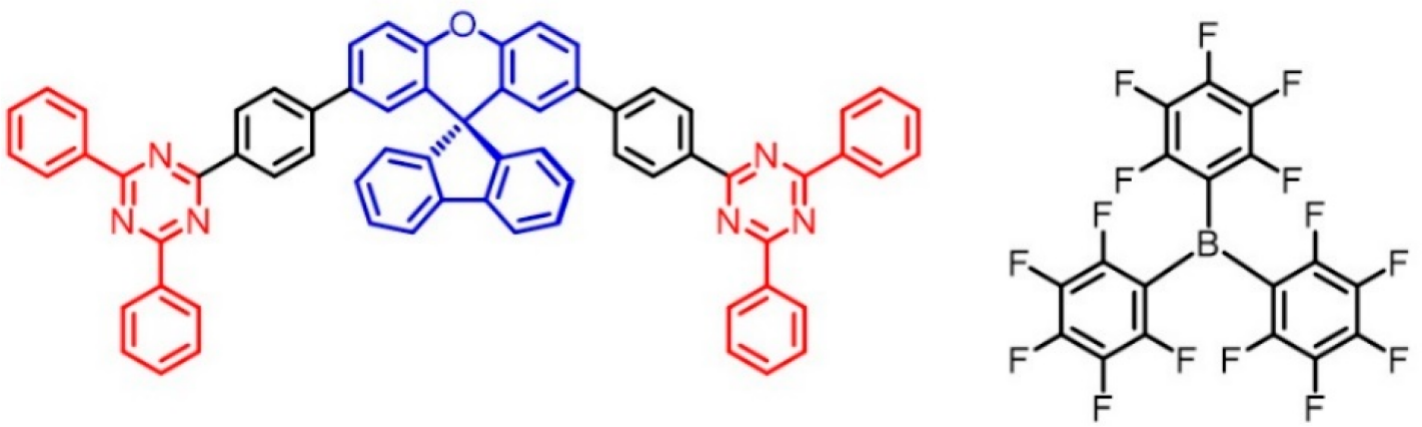
Molecular structures of *pTR*ZSX (left) and BCF (right).

## Results and Discussion

UV–visible absorption and fluorescence spectra were used to examine the formation of Lewis acid–base adducts, as shown in Figures 2a and 2b, respectively. With the addition of BCF to Lewis base *p*TRZSX, a new absorption peak appeared at around 475 nm, attributed to the formation of *p*TRZSX:BCF adducts. Compared with the neat *p*TRZSX film, the red-shifted absorption edge of the doped film indicates the reduction of the bandgap. Furthermore, *p*TRZSX exhibited an emission peak at about 418 nm in the neat film. In contrast, the fluorescent peak of the adduct film (50:50, w/w) was red-shifted to 598 nm, accompanied by the complete disappearance of the emission from *p*TRZSX itself. The inset of Figure 2b shows the PL images of the films spin-coated on the glass substrates under UV illumination, changing from deep-blue to orange-red. As shown in Figure 2c, with the increasing amount of BCF, the intensity of the emission from the adducts increased and saturated at a peak of 597 nm increases accordingly. Meanwhile, the deep-blue emission from *p*TRZSX decreased gradually.

**Figure 2:**
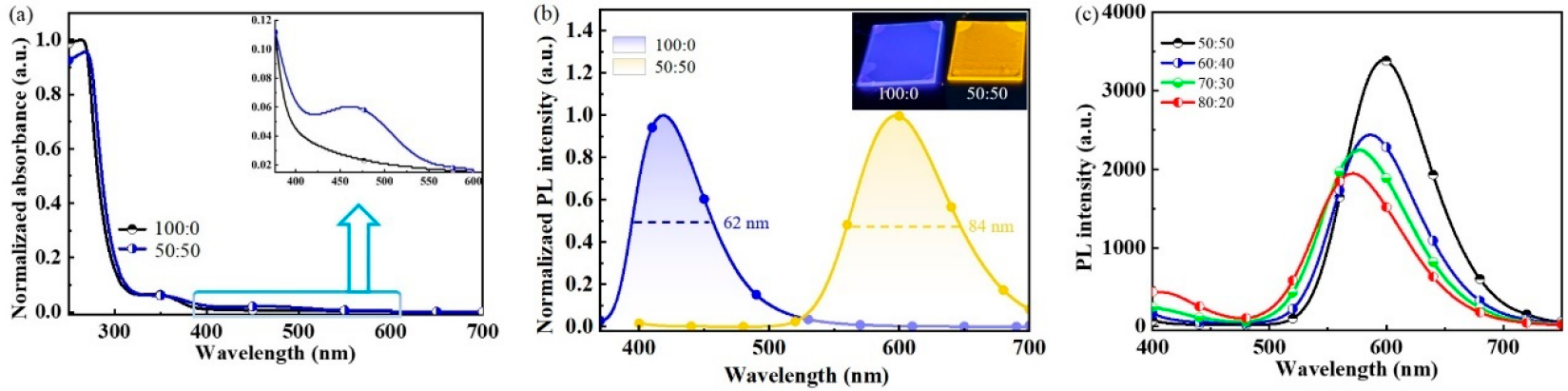
(a) UV–visible absorption and (b) normalized fluorescence spectra of the films with *p*TRZSX:BCF (100:0 and 50:50, w/w). Inset: photographs of the spin-coated films under UV (365 nm) illumination. (c) Fluorescence spectra of the films with different amounts of the Lewis acid BCF.

The adducts of pTRZSX:BCF = 1:1 (w/w) and neat pTRZSX in the solid-state film were investigated by X-ray photoelectron spectroscopy (XPS). As shown in Figures 3a and 3b, after the incorporation of BCF, a new nitrogen (N) 1s peak appeared at 402 eV, ascribed to the coordination of BCF with pTRZSX. The electrons on the triazine moieties were transferred to the boron atom, making the nitrogen atom more electron deficient. Therefore, the modification of the chemical environment of the nitrogen atom resulted in the increased binding energy.

**Figure 3:**
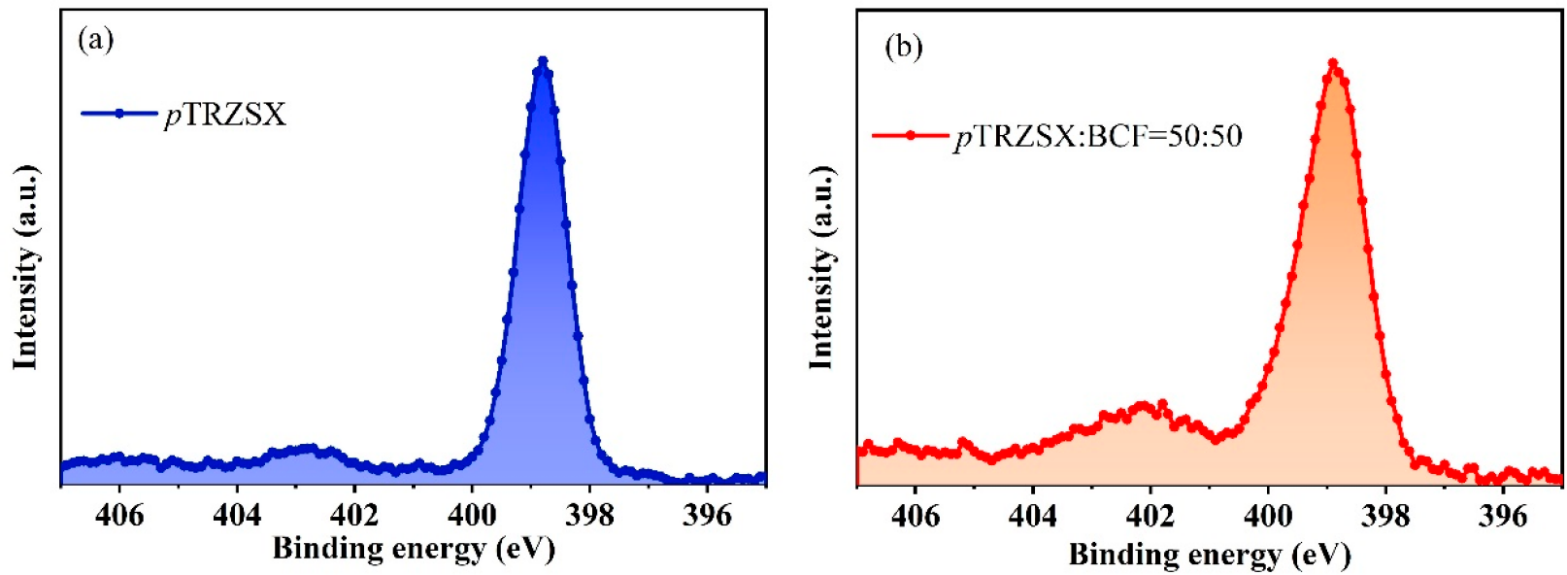
XPS spectra of nitrogen (N) 1s of (a) *p*TRZSX and (b) *p*TRZSX:BCF = 1:1 (w/w).

In order to testify the concept of decoupling the Lewis acid–base adduct with another Lewis base, i.e., erasing the encrypted information), triethylamine (TEA) was obtained after screening. Figure 5a presents the fluorescence spectra when different molar ratios of triethylamine (TEA) were added to the solutions of *p*TRZSX:BCF = 1:1 (w/w) before spin-coating. Once the amount of TEA increased, the orange-red emission from the adduct gradually disappeared. It is worth noting that the deep-blue emission of *p*TRZSX was fully recovered when the molar ratio of TEA:BCF reached 1:1. Therefore, it is reasonable that the coordination bond of the Lewis acid–base adduct based on *p*TRZSX:BCF was destroyed since the Lewis acid BCF was preferentially coordinated and neutralized with TEA. The inset of Figure 4a shows the PL images of the films spin-coated on the glass substrates under UV illumination. When the amount of TEA increased, the PL color of the film gradually recovered from orange-red to deep-blue.

**Figure 4:**
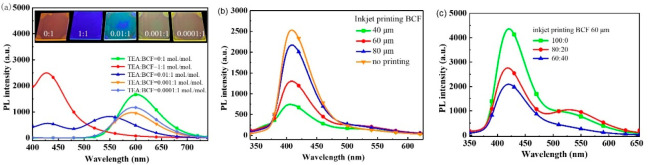
(a) Fluorescence spectra of the films *p*TRZSX:BCF = 1:1 (w/w) and different molar ratios of TEA relative to BCF (0.0001, 0.001, 0.1 and 1, respectively) added to *p*TRZSX:BCF. Inset: photographs of the spin-coated films under UV (365 nm) illumination. (b) Fluorescence spectra of the films after printing BCF (3 mg/mL, CB:DCM = 1:1) onto the seeding layer of *p*TRZSX with different interdot spacing (40, 60, and 80 μm, respectively). (c) Fluorescence spectra of the inkjet-printed films with different amount of PVK (0, 20, and 40 wt %, respectively) doped in the seeding layer (60 μm interdot spacing and ink: BCF, 3 mg/mL).

Inkjet printing provides a fast way to pattern the films. Moreover, the interdot spacing could modulate the coordination of the Lewis acid–base adducts and energy transfer. Therefore, we proposed and employed the BCF ink to pattern the deep-blue emissive *p*TRZSX film. It is expected that, when the interdot spacing is reduced, the emission intensity of *p*TRZSX would be correspondingly reduced, due to the increased amount of the adducts formed in the printed area. To modulate the morphology and prevent the seeding layer from easily dissolved by the ink during inkjet printing, the hydrophobic polymer poly­(*N*-vinylcarbazole) (PVK) was added to *p*TRZSX, serving as the seeding layer that was completely transparent under room light.

As confirmed in Figure 4b, the deep-blue emission was gradually quenched when reducing the interdot spacing during inkjet printing of BCF onto the seeding layer of *p*TRZSX. In order to obtain the efficient color tuning, PVK was doped into *p*TRZSX as the seeding layer. With the interdot spacing fixed at 60 μm, the emissive pattern could be feasibly modulated by changing the concentration of PVK in the seeding layer (see Figures 4c and S1).

As a proof-of-concept to protect the encrypted information from being easily decoded with UV light or from being removed completely, TEA was screened as the quencher or chemical eraser. It is found that the concentration of PVK in the seeding layer, which enhanced the solvent-resistant property, played a role in controlling the erasing efficiency of TEA (see Figure S1c). Due to the minimal interaction between PVK and BCF, incorporating PVK into the substrate allows for the easy and rational modulation of the interaction between BCF and *p*TRZSX. When the PVK content reached 40 wt %, the residual emission of the adducts was slightly visible. Nevertheless, an appropriate content of PVK can improve the film-forming properties and robustness of small molecules as the seeding layer. In contrast, all the patterns were completely removed with the low content of PVK in the seeding layer.

Based on inkjet printing of the Lewis acid BCF onto the seeding layer consisting of Lewis base *p*TRZSX, clear QR codes could be generated. Figures 5a and 5e exhibit the printed QR code images, which could be rapidly identified by smartphone. The microscope images shown in Figure S2 displayed the patterns printed with an interdot spacing of 80 μm. The Lewis acid–base adduct formation in the printed area was clearly demonstrated. Since the coordination bonds formed by Lewis acid–base adducts tend to be temperature sensitive, the encrypted information after inkjet printing could be eliminated at the elevated temperature (see Figures 5b and 5f). As shown in Figure 5b and 5f, the printed QR code patterns were totally invisible, demonstrating the feasibility of fast information erasing. The alternative approach to erase the encrypted information was successfully testified by spraying TEA (TEA:ethanol = 10:90, v/v) to neutralized BCF, as illustrated in Figures 5d and 5h, The printed QR code was faded with TEA spray. Although the pattern of the QR code was slightly visible in Figure 5d, the encrypted information essentially disappeared when PVK was introduced in the seeding layer (Figure 5h).

**Figure 5:**
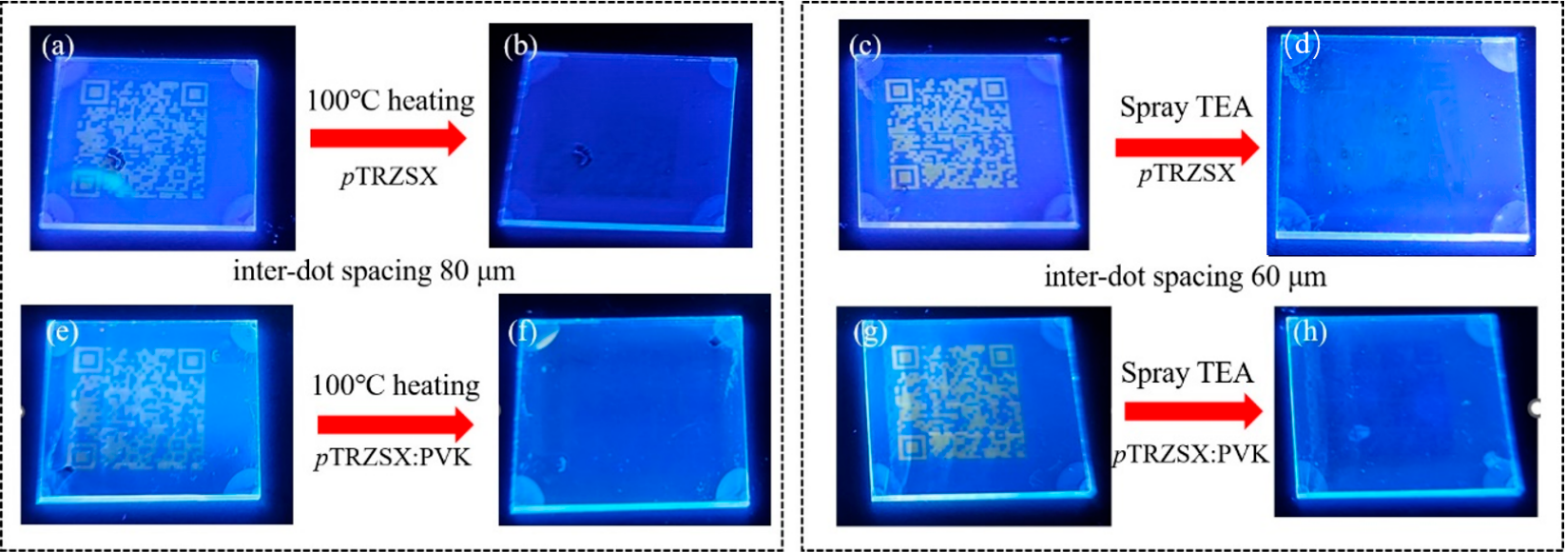
Images of the samples under UV illumination. (a) QR code after inkjet printing of BCF (interdot spacing: 80 μm) onto the seeding layer of *p*TRZSX and (b) following a heating process at 100 °C. (c) QR code after inkjet printing of BCF (interdot spacing: 60 μm) onto the seeding layer of *p*TRZSX and (d) after spraying TEA onto the sample. (e) QR code after inkjet printing of BCF (interdot spacing: 80 μm) onto the seeding layer of *p*TRZSX:PVK (80:20, w/w) and (f) followed a heating process at 100 °C. (g) QR code after inkjet printing of BCF (interdot spacing: 60 μm) onto the seeding layer of *p*TRZSX:PVK (80:20, w/w) and (h) after spraying TEA onto the sample.

## Conclusion

In summary, Lewis acid–base adducts and their chemistry were utilized to prove information encryption, considering their weak coordination bonds. A QR code can be quickly and precisely prepared by inkjet printing. The single-component Lewis acid as the ink was feasible to generate the luminescent patterns by changing the concentration and interdot spacing during inkjet printing. The inkjet printing with an interdot spacing of 60 μm achieved the best display quality and luminescence under UV excitation when inkjet-printed on the *p*TRZSX seeding layer. Meanwhile, the QR code can be effectively erased by heating at an elevated temperature or spraying a stronger Lewis base. A Lewis acid–base adduct provides an effective strategy toward advanced anticounterfeit with high security reliability, fast response, and ease of writing and erasing.

## Supplementary Material



## Notes

Material synthesis, inkjet printing procedure, photos of the samples, and microscopy images of the inkjet-printed films (PDF)
