# Supplementary material for: Writing and Erasing Encryption Information Based on Frustrated Lewis Pair Chemistry
Source: Precis Chem. 2023 Jul 28;1(7):418–22. doi: 10.1021/prechem.3c00062 (PMC12382413; doi:10.1021/prechem.3c00062)
Supplement: Supplementary file 1 [file pc3c00062_si_001.pdf]

Supporting Information

Writing and erasing encryption information based on Frustrated Lewis pair chemistry

Hui Fang[1], Xiao Long[2], Xia Lan[2], Bao-Yi Ren[2,*], and Guohua Xie[1,3*]

[1]Sauvage Center for Molecular Sciences, Hubei Key Lab on Organic and Polymeric Optoelectronic Materials, Department of Chemistry, Wuhan University, Wuhan 430072, China

[2]College of Science, Shenyang University of Chemical Technology, Shenyang 110142, China

[3]The Institute of Flexible Electronics (Future Technologies), Xiamen University, Xiamen 361005, China

*Corresponding email addresses: renbaoyi@syuct.edu.cn (B.R.) and guohua.xie@whu.edu.cn (G.X.).

Experimental Section

Synthesis of *p*TRZSX

(2',7'-bis(4-(4,6-diphenyl-1,3,5-triazin-2-yl) phenyl) spiro[fluorene-9,9'-xanthene]):*p*BrSX (0.40 g, 0.82 mmol), TRZ-Bpin (0.78 g, 1.80 mmol), tetrakis (triphenylphosphine)palladium(0) (0.05 g, 0.04 mmol) and K_2CO_3 (0.63 g, 4.59 mmol) were dissolved in H_2O/THF (12 mL, V_{H2O}:V_{THF} = 1:3) and the mixture was heated and stirred for 72 h at 80°C under nitrogen. The cooled reaction mixture was poured into water, extracted with dichloromethane, and dried over $MgSO_4$. The crude product was purified by silica column chromatography (DCM:PE = 1:1) to give the product as a white solid (0.15 g, 26%).

Inkjet printing of the luminescent Lewis acid-base adducts

The pre-patterned glass substrates were cleaned with acetone and ethanol, consecutively. Subsequently, *p*-TRZSX:(20 wt%) PVK was prepared by spin-coating directly onto the glass substrates. Then the BCF inks (3 mg/ml) were inkjet-printed with a desktop microelectronic printer (Prtronic, Shanghai) on the seeding layer with the different inter-dot spacing. The preheating temperature of the substrate was fixed at 40℃ during inkjet printing.

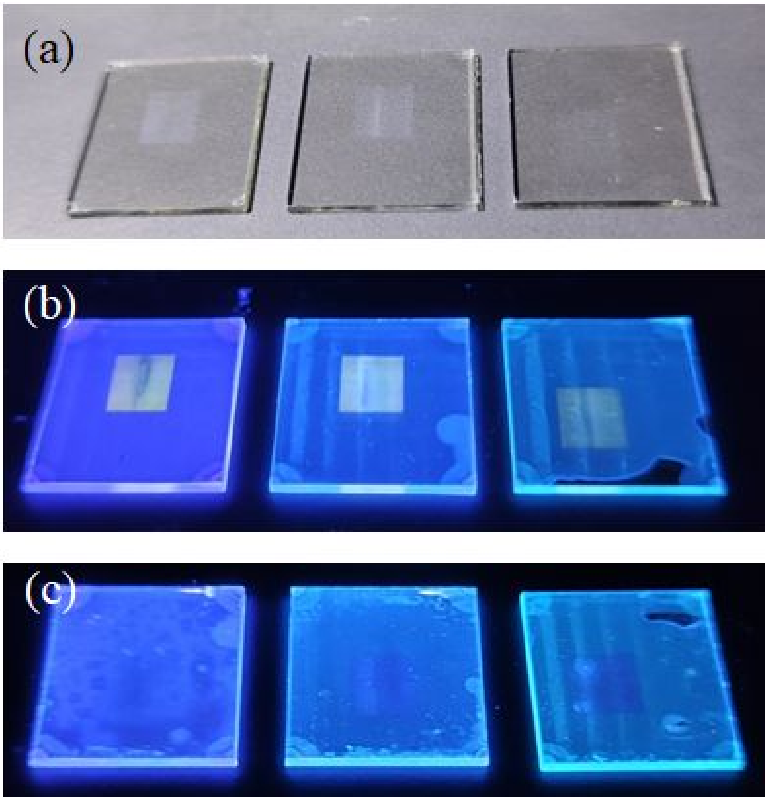

Figure S1. Photo of the sample with the seeding layer: *p*-TRZSX:PVK: (a) after inkjet printing of BCF under room light, (b) after inkjet printing of BCF under UV light, and (c) after TEA washing on the samples shown in (b). From left to right: 0 wt.%, 20 wt.%, and 40 wt.% of PVK, respectively doped in *p*-TRZSX as the seeding layer.

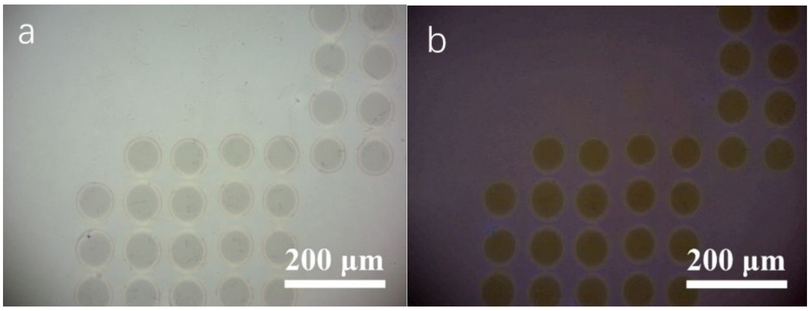

Figure S2. Microscopy images of the printed films (80 μm inter-dot spacing, ink: BCF 3 mg/ml, and seeding layer: *p*-TRZSX:PVK (80:20, wt./wt.) at the heating temperature of 40 ℃. (a) under room light and (b) under UV light (365 nm).
